# The association between entrapment and suicidality in adolescents with depressive disorders: a moderated mediation analysis involving depressive symptoms and gender

**DOI:** 10.3389/fpsyt.2026.1727843

**Published:** 2026-02-24

**Authors:** Hao Wu, Xin Chen, Weicui Tian, Yongchao Lin, Fajie Huang, Meiling Liao

**Affiliations:** 1 School of International Journalism and Communication, Beijing Foreign Studies University, Beijing, China; 2 School of Health, Fujian Medical University, Fuzhou, China; 3 School of Public Health and Health Management, Fujian Health College, Fuzhou, China; 4 Fuzhou Neuropsychiatric Hospital Affiliated to Fujian Medical University, Fuzhou, China

**Keywords:** adolescent depression, depressive symptoms, entrapment, gender differences, integrated motivational–volitional (IMV) model, suicidality

## Abstract

Preventing suicide among adolescents with depression remains a global health priority. Entrapment has been shown to be associated with suicidality, with depressive symptoms potentially accounting for part of this association, and these associations may vary by gender. To clarify these relationships, this study examined the statistical associations among entrapment, depressive symptoms, and suicidality, with particular attention to the statistical mediating role of depressive symptoms and the moderating role of gender. This study utilized a cross-sectional design, recruiting 406 adolescents with depressive disorders (79.6% female). Entrapment (Entrapment Scale), depressive symptoms (PHQ-9), and suicidality (SBQ-R) were assessed. Mediation and moderated mediation models (PROCESS macro, Models 4 and 8) were tested with age as a control variable, and bootstrapping procedures (5,000 resamples) were used to evaluate the robustness of the effects. Results indicated that (1): female participants reported significantly higher levels of entrapment, depressive symptoms, and suicidality than males (2); entrapment was positively associated with both depressive symptoms and suicidality (3); depressive symptoms statistically mediated the relationship between entrapment and suicidality; and (4) gender moderated the direct association, while the indirect (statistical mediation) association showed no significant gender differences. These findings suggest that the observed pattern of associations is consistent with theoretical models of suicidality, while also highlighting the importance of gender-sensitive clinical considerations: for males, clinical care should prioritize identifying and alleviating entrapment, whereas for females, comprehensive management addressing both entrapment and depressive symptoms may be more effective.

## 1 Introduction

Depression is one of the leading contributors to the global burden of disease and represents the most prevalent psychiatric disorder among children and adolescents, making it an urgent public health concern (1, 2). Epidemiological surveys indicate that between 2001 and 2020, the global prevalence of depressive symptoms among adolescents increased from 24.0% to 37.0%, reflecting both high prevalence rates and a trend toward earlier onset (3). Even more concerning, depression is the primary risk factor for suicide in adolescents, with affected individuals demonstrating a markedly elevated risk of suicidal behavior (4). The rate of suicide attempts among adolescents with depression can be up to five times higher than that of their peers without depression (4). Given that adolescence is a critical period of social, emotional, and cognitive development, the presence of depression coupled with suicidality during this stage poses severe risks to long-term mental health and psychosocial functioning. Therefore, identifying core psychological mechanisms associated with suicidality in adolescents with depressive disorders remains a pressing theoretical and clinical priority.

Recent research on suicide increasingly suggests that the core driving force behind an individual’s suicidal ideation may not be a genuine desire to die, but rather an urgent motivation to escape from experiences perceived as unbearable and inescapable (5). This motivational state is termed entrapment, defined as a cognitive–affective experience characterized by feelings of being trapped, powerless, and unable to escape from internal or external stressors. Entrapment is a unique, inescapable cognitive-affective state that represents a motivation to flee from pain, distinguishing it from the broader cluster of emotional, cognitive, and somatic symptoms characteristic of depression (6–10). Adolescents, who are in a developmental stage characterized by heightened emotional reactivity and ongoing cognitive maturation, may be particularly vulnerable to entrapment when confronted with academic stress, interpersonal conflict, or identity crises (11–13). Although robust associations between entrapment, depression, and suicidality have been documented in adult samples (14), far less is known about how these processes operate in clinically depressed adolescents, particularly with respect to potential mediating associations and the moderating role of gender.

In mainstream theories of suicide, entrapment is often regarded as a key factor in the emergence of suicidal ideation and behavior (10, 15, 16). The Integrated Motivational–Volitional (IMV) model of suicidal behavior posits that exposure to adverse experiences such as defeat, humiliation, or social exclusion can lead individuals to feel trapped when they are unable to resolve or escape these stressors, thereby activating suicidal motivation (15, 17). In this framework, entrapment functions as the core motivational mechanism linking experiences of defeat or humiliation to suicidal ideation (18). Empirical studies strongly support this proposition. For instance, Cramer et al. found that entrapment was positively associated with both suicidal ideation and behaviors in a large adult sample (19), while De Beurs et al. demonstrated that entrapment was independently associated with suicidal behavior across both clinical and community populations (20). Based on this theoretical and empirical foundation, we hypothesize that entrapment is positively associated with suicidality in adolescents with depression (H1).

Beyond its direct association with suicidality, entrapment may also be associated with suicidality indirectly through its correlation with depressive symptoms. Social Rank Theory (SRT) provides a complementary explanatory framework, suggesting that persistent experiences of failure, loss, or low social rank activate maladaptive defensive responses, resulting in feelings of entrapment and subsequent depressive affect (21). From this perspective, entrapment reflects the psychological experience of perceived defeat and low status, which may be associated with depressive symptoms that, in turn, heighten suicidality. Empirical findings are consistent with this theoretical proposition. For example, a longitudinal study by Alys Wyn Griffiths et al. found that baseline entrapment predicted worsening depressive symptoms over a 12-month period among formal caregivers (22). Similarly, Choi and Shin reported that entrapment not only directly correlated with depressive symptoms among Korean university students but also was indirectly associated with depression by heightening hopelessness (23). Although these studies suggest a potential temporal ordering, the present study focuses on examining statistical associations within a cross-sectional framework. Based on this evidence, we hypothesize that entrapment is positively associated with depressive symptoms (H2a).

Depressive symptoms, in turn, represent one of the most consistent and robust correlates of suicidality (24, 25). Several suicide theories highlight how depressive cognitions increase the recurrence of suicidal thoughts, which may eventually become entrenched cognitive tendencies that intensify suicidal ideation (26). Supporting this view, Zhou et al. in a study of 565 university students with major depressive disorder, found depressive symptoms to be the strongest direct correlate of suicidal ideation (25). Accordingly, we hypothesize that depressive symptoms are positively associated with suicidality (H2b). Taken together, these findings suggest that entrapment may be positively associated with suicidality both directly and indirectly, with depressive symptoms potentially accounting for part of this association. Given the cross-sectional nature of the present study, depressive symptoms are examined as a statistical mediator rather than as evidence of a confirmed causal or temporal mechanism. Therefore, we hypothesize that depressive symptoms statistically mediate the association between entrapment and suicidality (H2).

Gender may further shape the strength of these associations, acting as an important boundary condition (H3). Adolescence is a period during which gender-based socialization intensifies, and substantial gender differences have been documented in the expression of depression and suicidality. Specifically, this moderation is expected to manifest in two ways. First, regarding the relationship between entrapment and depressive symptoms, females may show a stronger association. Several studies have reported that adolescent females tend to experience higher levels of both entrapment (19, 27) and depressive symptoms (28). From a psychosocial perspective, females are generally more sensitive to interpersonal stress and tend to employ ruminative coping styles in response to negative emotions. This female-typical coping style (e.g., higher tendency for rumination) may be linked to the distressing experience brought about by entrapment being more easily internalized and amplified, potentially strengthening its association with depressive symptoms. Although some studies in clinical samples did not find gender differences in entrapment (29), gender socialization processes during adolescence may still contribute to variability in the strength of the entrapment - depression association. Accordingly, we hypothesize that gender moderates the association between entrapment and depressive symptoms (H3a). Second, gender may also moderate the direct relationship between entrapment and suicidality, with males demonstrating greater vulnerability. From the perspective of the IMV model and sociocultural frameworks, traditional masculinity norms—which emphasize emotional suppression and autonomous problem-solving—often stigmatize psychological help-seeking behavior. This societal expectation may prevent males experiencing high-intensity entrapment from utilizing social support or emotional disclosure, thus leaving the association between entrapment and suicidality unbuffered (17). In contrast, females are generally permitted more emotional expression and help-seeking behaviors through socialization, which may attenuate the direct entrapment–suicidality association. Therefore, we hypothesize that gender moderates the association between entrapment and suicidality, with the association being stronger in males (H3b). Collectively, this study will systematically examine whether gender moderates the strength of these two associations within a moderated mediation framework, with an emphasis on identifying gender-differentiated patterns of association rather than establishing gender-specific causal mechanisms.

**Figure 1:**
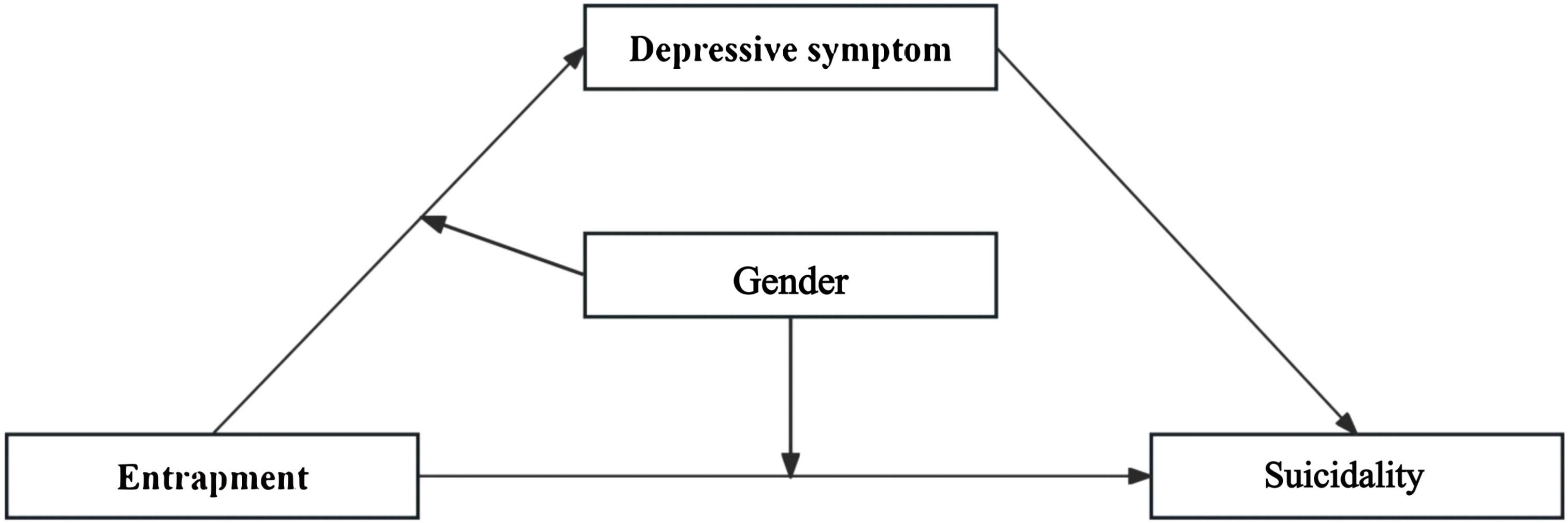
Conceptual model of the study design.

In summary, building upon the Integrated Motivational–Volitional model and Social Rank Theory, this study adopts a cross-sectional design to examine a moderated mediation model in clinically diagnosed adolescents with depression (see **Figure 1**). Specifically, we examine the statistical association between entrapment and suicidality (H1), explore whether depressive symptoms statistically account for part of this association (H2), and assess whether gender moderates the strength of both the entrapment - depressive symptoms association and the direct association between entrapment and suicidality (H3). The contributions of this research are threefold. First, unlike most previous studies focusing on community or non-clinical samples, this study utilizes a sample of clinically diagnosed adolescents with depression, lending higher clinical specificity and intervention guidance value to the findings. Second, by integrating gender as a moderating variable within an IMV-informed framework, this study examines whether the associations among entrapment, depressive symptoms, and suicidality differ by gender, thereby extending existing theoretical models while remaining consistent with the constraints of a cross-sectional design. Finally, the use of a brief entrapment scale facilitates the quick and effective identification of high-risk individuals in clinical practice. Overall, the findings are expected to elucidate the psychological factors associated with suicidality in adolescents with depression and provide empirical evidence for developing gender-specific clinical intervention strategies.

## 2 Materials and methods

### 2.1 Participants

This study was approved by the Ethics Committee of Fuzhou Second General Hospital, Institute of Neurological and Psychiatric Prevention and Treatment (Approval No.: 2025-06). From December 2024 to April 2025, adolescents diagnosed with depressive disorders were consecutively recruited from the outpatient psychological or psychiatric clinics of the Neuropsychiatric Prevention and Treatment Institute of Fuzhou Second General Hospital and Longyan Third Hospital. Both institutions are tertiary-level public hospitals in mainland China, including specialized psychiatric services, and operate standardized systems for the assessment and treatment of adolescent mental disorders.

All participating adolescents with depressive disorders were clinically diagnosed by licensed psychiatrists who had received systematic training in the diagnosis and treatment of adolescent psychiatric disorders, in accordance with the diagnostic criteria of the Diagnostic and Statistical Manual of Mental Disorders, Fifth Edition (DSM-5). Diagnoses were made following routine clinical diagnostic procedures and comprehensive clinical judgment, including clinical interviews and review of medical records. No additional structured or semi-structured diagnostic interviews were administered specifically for research purposes.

The inclusion criteria were as follows (1): a clinical diagnosis of depressive disorder according to DSM-5 criteria made by a psychiatrist (2); age between 11 and 18 years (3); absence of severe psychotic symptoms (e.g., hallucinations or delusions) or cognitive impairments (e.g., intellectual disability or major brain injury) (4); voluntary participation with written informed consent obtained from both the participant and their legal guardian; and (5) ability to independently complete the questionnaire.

With respect to comorbidities, adolescents with common comorbid conditions such as anxiety disorders were not excluded, in order to enhance the representativeness of the sample to real-world clinical settings. However, individuals with a history of bipolar disorder, psychotic disorders, or other severe neuropsychiatric conditions were excluded based on clinical evaluation and medical records. These criteria were intended to ensure diagnostic accuracy and consistency while minimizing potential confounding factors. All questionnaires were completed in designated assessment rooms within the hospitals and were administered individually under the guidance of trained psychology undergraduate or graduate students with formal instruction in psychological assessment.

Ultimately, this study recruited 407 adolescent patients who met the inclusion criteria. One participant withdrew mid-study due to loss of interest in continuing participation, resulting in a final sample of 406 valid participants (with an effective response rate of 99.75%). The participants had a mean age of 15.22 ± 1.92 years; 82 were male (20.4%) and 324 were female (79.6%), reflecting the higher prevalence of depression among adolescent females observed in epidemiological studies. According to common psychometric sampling principles—typically requiring at least ten participants per item—a minimum of 170 participants was needed to ensure adequate statistical power for the analyses, given that the three scales used in this study comprised a total of 17 items. The final sample size of 406 thus provided sufficient statistical power to support subsequent structural modeling and association analyses.

Descriptive statistics were calculated based on commonly used cut-off criteria for depressive symptom severity (PHQ-9) (30) and suicidality risk (SBQ-R) (31). Regarding depressive symptom severity, 31 participants (7.6%) reported minimal symptoms (0 – 4), 47 (11.6%) mild symptoms (5 – 9), 70 (17.2%) moderate symptoms (10 – 14), 115 (28.3%) moderately severe symptoms (15 – 19), and 143 (35.2%) severe symptoms (20 – 27). With respect to suicidality (using a cut-off score of 7), 79 participants (19.5%) reported no clinically significant suicidality, whereas 327 participants (80.5%) exhibited clinically significant levels of suicidality.

### 2.2 Instruments

#### 2.2.1 Four-item entrapment scale

The Entrapment Scale was originally developed by Gilbert and Allan and consists of 16 items assessing two dimensions: Internal Entrapment (IE) and External Entrapment (EE) (32). The scale has been translated into multiple languages and has demonstrated good reliability and validity across cultures. Building on this foundation, Huang et al. following prior research (20), developed and validated a brief version of the Entrapment Scale specifically for adolescents with depressive disorders in the Chinese cultural context. The short form comprises four items, maintaining the two-dimensional structure: IE and EE. The IE dimension includes two items: “I have a strong desire to escape from things in my life” and “I often feel like I would just like to run away.” The EE dimension also includes two items: “I feel powerless to change myself” and “I feel I am in a deep hole that I can’t escape.” Each item is rated on a 5-point Likert scale ranging from 0 (“Not at all like me”) to 4 (“Very much like me”). The total score is obtained by summing the four items, with higher scores indicating greater psychological entrapment. Although the brief ES comprises fewer items, its design and validation were specifically tailored for use with clinically diagnosed adolescent patients with depression (20). The short form has been empirically demonstrated to exhibit appropriate structural validity, reliability, and criterion validity comparable to the original 16-item scale. Crucially, it effectively captures the construct of entrapment within this population and is reliably associated with related outcomes such as depression, anxiety, and suicidal ideation. Therefore, this brief scale is highly suitable for use with the clinical sample in the present study, offering advantages in rapid assessment within clinical practice. In the present study, the Cronbach’s α coefficient for the total short-form ES was 0.89, while the α values for the IE and EE subscales were 0.88 and 0.80, respectively, indicating excellent internal consistency reliability.

#### 2.2.2 Patient Health Questionnaire

The Patient Health Questionnaire-9 (PHQ-9) was developed by the U.S. National Institute of Mental Health (NIMH) based on the diagnostic criteria for depressive disorder outlined in the Diagnostic and Statistical Manual of Mental Disorders, Fourth Edition (DSM-IV) (33). The scale includes nine items (e.g., “Trouble falling or staying asleep, or sleeping too much”), each rated on a 4-point Likert scale ranging from 0 (“Not at all”) to 3 (“Nearly every day”). The PHQ-9 has been widely used in both clinical and research contexts and has demonstrated strong cross-cultural adaptability, reliability, and validity. It has also been extensively used in Chinese populations as a self-report measure of depressive symptoms (30). The total score is obtained by summing all items, with higher scores indicating greater severity of depressive symptoms. For the purpose of the present study, the PHQ-9 total score was used as a continuous variable index of depressive symptom severity in the analyses. Using a continuous variable is statistically more appropriate than a dichotomous cut-off score when examining complex models such as mediation and moderation, as it preserves maximum variance and statistical power in the hypothesized association. In the present study, the Cronbach’s α coefficient was 0.87, indicating good internal consistency reliability.

#### 2.2.3 Suicidal Behaviors Questionnaire-Revised

The Suicidal Behaviors Questionnaire-Revised (SBQ-R) was developed by Osman et al. and consists of four items designed to assess suicidality. It has been widely applied in Chinese populations and has demonstrated good reliability and validity (31). The first item evaluates lifetime suicidal ideation or attempts; the second assesses the frequency of suicidal thoughts during the past 12 months; the third measures the occurrence of suicide threats; and the fourth assesses the likelihood of future suicidal behavior. The SBQ-R uses a multiple-choice response format, with total scores calculated by summing all items. Total scores range from 3 to 18, with higher scores indicating greater suicidality. The SBQ-R total score is considered a psychometrically grounded composite index that quantifies the overall severity of an individual’s suicidality (31). Consistent with our analytical strategy for other measures, the SBQ-R total score was utilized as a continuous variable index of suicidality in the present study. This approach is statistically preferred for mediation and moderation analyses, as using a continuous measure effectively preserves the full range of variability and statistical power. In the current study, the Cronbach’s α coefficient was 0.79, suggesting acceptable internal consistency reliability.

### 2.3 Data analysis

Data entry and preliminary analyses were conducted using SPSS 21.0, including exploratory factor analysis, descriptive statistics, and correlation analyses. Confirmatory factor analysis (CFA) was performed with Mplus 8.0 to examine potential common method bias among the study measures. In addition, mediation and moderation analyses were conducted using Hayes’s PROCESS macro (version 3.2). Independent, dependent, mediating, and moderating variables were entered into the respective models, and the significance and confidence intervals of effects were estimated using bias-corrected bootstrap procedures. Prior to running the PROCESS models, all variables were z-standardized, and predictor variables were mean-centered before constructing interaction terms. All bootstrap procedures were based on 5,000 resamples.

## 3 Results

### 3.1 Common method bias test

To examine the potential impact of common method bias, Harman’s single-factor test was first conducted. The unrotated exploratory factor analysis extracted four factors with eigenvalues greater than 1, with the first factor accounting for 40.74% of the total variance. This value is below the 50% threshold suggested by Podsakoff and Organ (34), indicating that common method bias was unlikely to pose a serious concern in this study.

Furthermore, a confirmatory factor analysis (CFA) was performed to compare the model fit between a single-factor model (in which all items loaded onto one latent factor) and a three-factor model (in which items for entrapment, depressive symptoms, and suicide risk loaded onto their respective latent factors) (35). As shown in **Table 1**, the single-factor model exhibited poor fit across all indices, whereas the three-factor model demonstrated significantly better fit (Δχ^2^ = 374.19, Δ*df* = 3, *p* < 0.001), with fit indices approaching acceptable standards. These results suggest that common method bias is not a serious threat to the validity of the study’s findings. Despite the evidence from these statistical tests suggesting that common method bias is not severe, it is important to acknowledge that both Harman’s single-factor test and the CFA model comparison method have inherent limitations. Therefore, common method bias is still acknowledged as a methodological limitation in this study.

**Table 1:** Comparison of fit indices between the single-factor and three-factor models.

Model	χ^2^	*df*	χ^2^/*df*	RMSEA	CFI	TLI	SRMR
Single-factor model	891.16	119	7.49	0.19	0.78	0.75	0.08
Three-factor model	516.97	116	4.46	0.09	0.89	0.87	0.06

### 3.2 Descriptive statistics and correlation analysis

Descriptive statistics and Pearson correlation analyses were conducted for gender, age, entrapment, depressive symptoms, and suicidality among adolescents with depression(see **Table 2**). The results indicated that the three core psychological variables—entrapment, depressive symptoms, and suicidality—were all significantly and positively correlated with one another.

**Table 2:** Descriptive statistics and correlation analysis (*n* = 406).

Variable	*M* ± *SD*	1	2	3	4	5
1 Gender	0.79 ± 0.40	–				
2 Age	15.22 ± 1.92	-0.04	–			
3 Entrapment	9.66 ± 4.73	.19^***^	-0.06	–		
4 Depressive Symptom	15.88 ± 6.64	.21^***^	-.14^**^	.73^***^	–	
5 Suicidality	10.45 ± 4.67	.20^***^	-0.07	.52^***^	.50^***^	–

*p* < 0.01 (^**^), *p* < 0.001 (^***^).

Gender (coded as 0 = male, 1 = female) was positively correlated with entrapment, depressive symptoms, and suicidality, suggesting that female patients scored significantly higher than males on all three variables. In addition, age was negatively correlated with depressive symptoms. Therefore, age was included as a control variable in the subsequent mediation and moderation analyses.

### 3.3 Indirect association between entrapment and suicidality via depressive symptoms

Entrapment was entered as the independent variable, suicidality as the dependent variable, depressive symptoms as the mediating variable, and age as the control variable. The indirect (mediated) association was tested using Model 4 of Hayes’s PROCESS macro.

As shown in **Table 3**, entrapment was significantly and positively associated with suicidality (β = 0.11, *t* = 12.22, *p* < 0.001) and depressive symptoms (β = 0.15, *t* = 21.13, *p* < 0.001). After including depressive symptoms in the model, both entrapment (β = 0.07, *t* = 5.52, *p* < 0.001) and depressive symptoms (β = 0.26, *t* = 4.20, *p* < 0.001) remained significantly correlated with suicidality.

**Table 3:** Mediation association analysis of depressive symptoms between entrapment and suicidality.

Variable	Suicidality			Depressive symptom			Suicidality		
	β	SE	*t*	β	SE	*t*	β	SE	*t*
Entrapment (X)	0.11	0.01	12.22^***^	0.15	0.01	21.13^***^	0.07	0.01	5.52^***^
Depressive Symptom							0.25	0.06	4.20^***^
Control variable									
Age	-0.02	0.02	-0.88^**^	-0.05	0.02	-2.86^**^	-0.01	0.02	-0.29
*R* ^2^	0.28			0.54			0.31		
*F*	75.98^***^			232.02^***^			58.65^***^		

β represents standardized regression coefficients, *p* < 0.01 (^**^), *p* < 0.001 (^***^).

A bootstrapping method with 5,000 resamples was employed to evaluate the statistical significance of the indirect association. As shown in **Table 4**, the 95% confidence interval (CI) for the indirect effect was [0.02, 0.06], which did not include zero, indicating a statistically significant indirect association between entrapment and suicidality through depressive symptoms. The indirect effect accounted for 36.36% of the total effect.

**Table 4:** Summary of total, direct, and indirect effects.

Effect type	Effect value	Boot SE	95% CI	Proportion of total effect (%)
Direct Effect	0.07	0.01	0.05, 0.10	63.64
Indirect Effect	0.04	0.01	0.02, 0.06	36.36
Total Effect	0.11	0.01	0.09, 0.13	

### 3.4 Moderating role of gender in the indirect association between entrapment and suicidality

To examine whether gender moderates the mediation association model described above, age was included as a control variable, and Model 8 of Hayes’s PROCESS macro was employed. As shown in **Table 5**, the interaction term between entrapment and gender was not significantly associated with depressive symptoms (β = -0.08, *t* = -1.06, *p* > 0.05), but it was significantly associated with suicidality (β = -0.23, *t* = -2.25, *p* < 0.05). These results suggest that gender does not moderate the association between entrapment and depressive symptoms, but it does significantly moderate the strength of the association between entrapment and suicidality.

**Table 5:** Moderation test of gender on the mediation association.

Variable	Depressive symptom			Suicidality		
	β	SE	*t*	β	SE	*t*
Entrapment (X)	0.77	0.07	10.62^***^	0.51	0.10	5.06^***^
Gender(MO)	0.15	0.09	1.73	0.16	0.11	1.47
X ×MO	-0.08	0.08	-1.06	-0.23	0.10	-2.25^*^
Depressive Symptom				-0.24	0.06	3.88^***^
Age	-0.05	0.02	-2.84^**^	-0.01	0.02	-0.37
*R* ^2^	0.54			0.32		
*F*	118.33^***^			37.71^***^		

β represents standardized regression coefficients, *p* < 0.05 (^*^), *p* < 0.01 (^**^), *p* < 0.001 (^***^).

**Figure 2:**
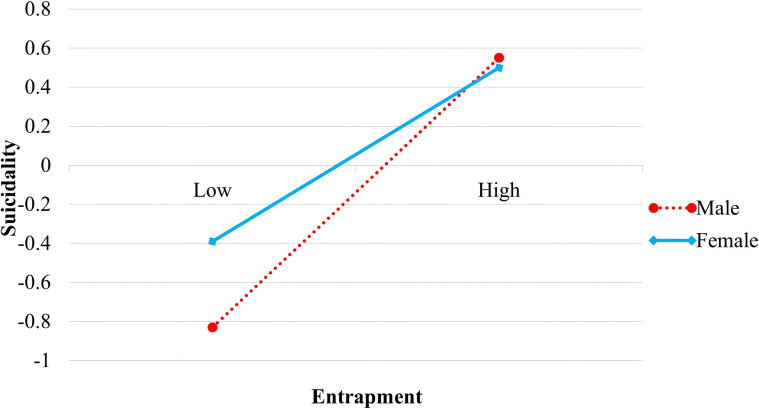
Moderating role of gender in the association between entrapment and suicidality.

To further interpret the moderated mediation association model, a simple slope analysis was conducted on the interaction between entrapment and gender. Entrapment was divided into high and low groups (± 1 SD from the mean), and interaction plots were generated for males and females (see **Figure 2**). The results showed that entrapment was significantly associated with suicidality for both males (β_simple slope_ = 0.51, *t* = 5.06, *p* < 0.001) and females (β_simple slope_ = 0.28, *t* = 4.45, *p* < 0.001), crucially, strength of this association was significantly greater in males than in females (β = -0.23, *t* = -2.25, *p* < 0.05).

Furthermore, subgroup analyses were conducted to examine the indirect associations across genders. Among males, the indirect association of depressive symptoms was significant (Effect 1 = 0.18, SE = 0.05, 95% CI: [0.08, 0.29]). Among females, the indirect association was also significant (Effect 2 = 0.16, SE = 0.04, 95% CI: [0.08, 0.25]). The difference between the two indirect association was not statistically significant (Effect 1 - Effect 2 = 0.02, 95% CI: [-0.07, 0.02]).

Taken together, these findings indicate that entrapment is statistically associated with suicidality both directly and indirectly through depressive symptoms, with depressive symptoms partially accounting for this association. Importantly, in this particular sample, gender was found to moderate the strength of the direct association between entrapment and suicidality, such that the association is stronger among male adolescents, whereas the indirect (mediated) association via depressive symptoms does not differ significantly by gender. This provides differentiated insights into the association underlying suicidality among male and female adolescents with depression.

## 4 Discussion

The present study aimed to integrate the IMV model and SRT to explore the dual associations linking entrapment and suicidality, and to examine the moderating role of gender in these associations. Our findings confirmed hypotheses H1 and H2, revealing that entrapment is significantly associated with suicidality through both a direct association and an indirect association involving depressive symptoms. Furthermore, the results supported Hypothesis H3b, showing that gender significantly moderates the direct association between entrapment and suicidality, while the indirect association via depressive symptoms remained stable across genders (Hypothesis H3a was not supported). The following sections detail the primary findings and discuss their theoretical and clinical implications.

### 4.1 Dual associations of entrapment and suicidality among adolescents with depression

The present study found that entrapment exhibited a significant positive association with suicidality in adolescents with depression, validating Hypothesis H1, which is highly consistent with a substantial body of prior research (19, 36, 37). For instance, Siddaway et al. conducted a meta-analysis encompassing 40 studies and reported a stable positive correlation between entrapment and both suicidal ideation and suicidal behavior (38). The current findings extend this evidence to a clinical sample of adolescents with depression, reinforcing the theoretical expectations of the Integrated Motivational–Volitional (IMV) model of suicidal behavior. Specifically, when individuals experience failure, humiliation, or social exclusion and lack effective emotion regulation capacities, they are more likely to report intense feelings of entrapment that are closely linked to suicidal motivation (15, 17). Consistent with De Beurs et al., who identified entrapment as a key correlate of suicidality (20), our findings provide strong empirical evidence of its associative role among adolescents with depression.

Importantly, the study further revealed that entrapment was not only directly associated with suicidality but also was indirectly associated with it through the mediating role of depressive symptoms, thus supporting H2. According to the Social Rank Theory (SRT), when individuals are chronically trapped in situations perceived as inescapable or uncontrollable, adaptive defensive responses may deteriorate into maladaptive psychological states, manifesting as entrapment, which is theoretically linked to elevated depressive symptoms (6). Depressive symptoms, through the sustained presence of depressive thought processes, may render suicidal ideation more salient within an individual’s mental experience, thereby strengthening its association with suicidality. Consequently, depressive symptoms play a crucial mediating role in the association between entrapment and suicidality.

Taken together, based on cross-sectional data, this study is the first to integrate the IMV model and SRT in a clinical sample of adolescents with depression. The findings support a dual-association framework in which entrapment is related to suicidality through both a direct association and an indirect association involving depressive symptoms. Specifically, this underscores the importance of a dual-target intervention strategy—simultaneously addressing entrapment-related distress and alleviating depressive symptoms—to effectively mitigate suicidality among adolescents with depression.

Despite the fact that the Dual-Association Framework of Entrapment proposed in the present study is highly consistent with the theoretical expectations of both the IMV model and SRT, it is important to explicitly acknowledge the limitations inherent in the use of cross-sectional data for causal inference. Specifically, although the present findings reveal significant associations among entrapment, depressive symptoms, and suicidality, the data do not allow for the determination of whether experiences of entrapment temporally precede increases in depressive symptoms and suicidality, or whether more severe depressive symptoms and pre-existing suicidal tendencies, in turn, intensify individuals’ perceptions of entrapment. Accordingly, the Dual-Association Framework of Entrapment examined in this study should be regarded as a theoretically informed, hypothesis-generating account of the potential relationships among these variables rather than as evidence of confirmed causal pathways. Clarifying the precise temporal ordering and causal mechanisms underlying these associations will require future research employing longitudinal designs or experimental approaches.

### 4.2 Gender as a moderator in the association between entrapment and suicidality among adolescents with depression

Another notable finding observed in this particular sample is that gender appeared to be associated with differential patterns within the dual-association framework linking entrapment and suicidality. It must also be explicitly stated that this study did not find a significant moderating effect of gender on the entrapment - depressive symptoms association (Hypothesis H3a was not supported).

Firstly, the direct association between entrapment and suicidality was observed to be stronger among male adolescents with depression than among females in this particular sample. This finding is consistent with Hypothesis H3b and is likely attributable to the shaping influence of prevalent gender norms on male behavior, particularly within the Chinese adolescent context (39). Specifically, traditional Chinese culture and social norms often emphasize masculine stoicism (“A man sheds tears only when his cause is truly lost”), along with requirements for autonomy and emotional restraint. These expectations result in males being less likely to express psychological vulnerability and more resistant to seeking professional psychological help (40–42). In practice, such patterns could weaken perceived social support and, in turn, potentially strengthen the association between psychological distress and suicidality (43, 44). For instance, Cleary conducted a qualitative study involving 52 young Irish men with histories of suicide attempts and found that participants who deliberately concealed their suicidal thoughts often engaged in self-medication through alcohol and strongly rejected peer or professional assistance (43). In a subsequent follow-up of hospitalized male suicide attempters, Cleary further confirmed that individuals adhering more strongly to traditional masculine norms—marked by emotional suppression and help-seeking avoidance—exhibited higher suicidality and greater recurrence of suicidal behavior (44). Thus, during gender socialization processes that valorize emotional control and self-reliance, male patients may suppress emotional expression and help-seeking behaviors when facing high levels of entrapment, thereby showing a stronger direct association between entrapment and suicidality.

Second, this study found that the mediating role of depressive symptoms between entrapment and suicidality was consistent across genders, with no significant group differences. This suggests that the indirect association may reflect a gender-invariant process. One possible explanation is that this “gender-invariant” pattern reflects deeper, evolutionarily conserved biological processes that transcend sociocultural boundaries. According to Social Rank Theory (SRT), low mood and submissive behavior are involuntary adaptive responses to social defeat, functioning to reduce aggression and signal submission to mitigate further conflict (45, 46). When such adverse situations persist, experiences of entrapment are commonly accompanied by sustained stress responses, which have been theoretically associated with neuroendocrine changes and depressive symptomatology (47). This stress–depression linkage is considered to be relatively universal and may operate similarly across genders, independent of sociocultural gender roles.

It is noteworthy that gender did not moderate the association between entrapment and depressive symptoms, contrary to Hypothesis H3a. As discussed above, this nonsignificant finding may reflect the broadly shared biological and emotional processes underlying depressive symptomatology. Additionally, because the current sample comprised clinically diagnosed adolescents with depression, the generally high baseline levels of depressive symptoms may have reduced the detectability of gender-specific effects.

Taken together, these considerations offer a plausible interpretive framework for the observed gender differences in this sample, rather than definitive evidence of distinct gender-specific mechanisms: in the process through which entrapment is positively associated with suicidality, the direct association appears to show a pattern of greater sensitivity among males in this sample, whereas the indirect association mediated by depressive symptoms shows cross-gender stability. This suggests that, although male and female adolescents with depression may differ in their behavioral manifestations of psychological pain and suicidal behavior, they nonetheless share similar underlying emotional and psychophysiological correlates. Such insights highlight the importance of adopting gender-informed yet mechanistically grounded approaches in adolescent suicide risk research and prevention.

### 4.3 Clinical implications

The findings of this study provide several targeted implications for the clinical management of adolescent depression.

First, entrapment should be identified as a core intervention target to potentially reduce its strong association with suicidality. In clinical practice, attention should extend beyond conventional monitoring of depressive affect to the proactive identification and management of patients’ experiences of entrapment. During the assessment phase, standardized tools such as the Entrapment Scale can be integrated with structured clinical interviews to systematically explore the cognitive and emotional core of the “no way out” experience. During the intervention phase, therapists can employ a cognitive-behavioral framework to guide patients in maintaining an “entrapment diary,” which helps them recognize automatic helpless thoughts triggered by specific situations and challenge absolute beliefs through cognitive restructuring techniques. Concurrently, integrating problem-solving training—following the standardized sequence of problem analysis, solution generation, action planning, and outcome evaluation—can enhance patients’ sense of coping efficacy when facing real-life stressors, thereby potentially attenuating the direct association between entrapment and suicidality.

Second, the stable mediating role of depressive symptoms underscores the importance of consolidating and optimizing conventional depression management to address this key indirect association. Clinicians should establish a dynamic symptom-monitoring system to track fluctuations in core symptoms such as low mood, self-denigration, and anhedonia. Reliable, validated instruments such as the Beck Depression Inventory can be administered routinely for this purpose. Therapeutically, contemporary approaches such as metacognitive therapy may be adopted to help patients develop awareness of and modify maladaptive responses to negative thoughts. Alternatively, rumination-focused cognitive-behavioral therapy can be used to target maladaptive repetitive thinking through attention refocusing and contextual reframing. Effective alleviation of depressive symptoms may thus help weaken the indirect association through which entrapment is linked to suicidality.

Finally, clinical interventions should adopt gender-sensitive strategies to achieve precision treatment. For male patients, interventions should focus on reducing help-seeking barriers by designing action-oriented, brief therapeutic models. For instance, translating abstract emotional work into concrete, skill-based “problem-solving steps” and using scenario-based exercises can improve engagement and reduce the perceived stigma around help-seeking. For female patients, interventions should emphasize integrative approaches that simultaneously address entrapment and depressive symptoms. Mindfulness-based training can be used to reduce emotional sensitivity, while cognitive restructuring involving family members can help minimize ruminative thinking. Together, these strategies create a comprehensive protective network encompassing cognitive, emotional, and behavioral dimensions.

Collectively, these clinical applications highlight the need for a dual-focus, gender-informed approach: targeting both entrapment and depressive symptoms, while tailoring intervention delivery to gender-specific psychological characteristics. Such a framework may contribute to improving the effectiveness and precision of suicide prevention efforts among adolescents with depression.

### 4.4 Research integration, limitations and future directions

The present study adopted a cross-sectional design in a clinical adolescent sample to, for the first time, integrate the IMV model (from entrapment to suicidal motivation) and SRT (from entrapment to depressive symptoms), revealing a “dual-association” framework linking entrapment and suicidality, and examining it through a gender-specific lens. Our results indicate that the direct association between entrapment and suicide risk varies by gender, potentially reflecting differences in gender norms and help-seeking behaviors; conversely, the indirect association involving depressive symptoms demonstrates cross-gender consistency, suggesting a shared psychophysiological foundation.

Future research should build upon this foundation by utilizing longitudinal designs to further validate the dynamic changes and potential causal relationships over time within these gender-differentiated mechanisms. Gender should be incorporated as a key element throughout the entire theoretical framework of suicidality, fostering the development of integrated models (IMV + SRT + Gender Perspective) that are more refined, culturally adapted, and rooted in biopsychosocial depth, thereby enhancing the understanding of the complexity of suicidality.

Despite its theoretical and practical contributions, this study has several limitations that should be addressed in future research.

Firstly, the cross-sectional design limits causal inference; therefore, longitudinal or experimental studies are needed to further validate the temporal relationships among entrapment, depressive symptoms, and suicidality (48).

Secondly, the present study is characterized by a gender imbalance in the sample, with a predominance of female participants. Although this distribution may, to some extent, reflect the gender patterns observed in clinical diagnoses of adolescent depression, the relatively small number of male participants (n = 82) may limit the stability and statistical power of parameter estimates within this subgroup. Accordingly, the observed moderating role of gender should be interpreted as preliminary and exploratory, and future research is needed to replicate these findings in more representative and gender-balanced samples (49).

Thirdly, the data primarily relied on self-report questionnaires. On the one hand, this raises the potential concern of common method bias (CMB). Although statistical controls like Harman’s single-factor test and CFA comparisons were applied, suggesting the bias is not severe, the inherent limitations of these statistical methods mean that systematic error introduced by the measurement approach cannot be entirely ruled out. On the other hand, self-report may be influenced by social desirability or concealment, potentially affecting the associations among entrapment, depressive symptoms, and suicidality. Therefore, future research should incorporate multimodal assessments, such as combining qualitative interviews (50), behavioral observation, or neuroendocrine indicators (e.g., cortisol levels), to enhance the ecological validity and objectivity of the findings.

Finally, to construct a more comprehensive theoretical model, future research should incorporate additional protective and risk factors, such as social support and emotion regulation strategies, to further dissect the psychosocial mechanisms underlying gender differences. To enhance the replicability and clinical relevance of the findings, future studies should additionally employ structured or semi-structured diagnostic interviews for research purposes and systematically record and control for more detailed clinical information, including the duration of depressive episodes, comorbid conditions (e.g., comorbid anxiety disorders), as well as more fine-grained indicators of symptom severity and clinical context.

## 5 Conclusions

Based on a cross-sectional study design, this study systematically investigated the mechanisms of the association between entrapment and suicidality in adolescents with depression. The findings indicate that entrapment is associated with suicidality both directly and positively, while also maintaining an indirect association through the mediation of depressive symptoms, forming a dual-association framework within this clinical sample. Specifically, in this sample, the direct association between entrapment and suicidality was observed to be stronger among males than females, whereas the indirect association via depressive symptoms demonstrated cross-gender universality. Although these findings require cautious interpretation and replication in larger and more gender-balanced samples, the results suggest that clinical interventions should prioritize the assessment and treatment of entrapment while adopting gender-informed strategies: for males, the focus should be on intervening in the entrapment experience to alleviate its direct association with suicidality; for females, comprehensive management of both entrapment and depressive symptoms is required to fully mitigate suicidality. It should be emphasized that these conclusions are based on cross-sectional correlational data, and future longitudinal studies are required to further confirm the dynamic changes and causal relationships within these associated pathways.

## Data Availability

The original contributions presented in the study are included in the article/**Supplementary Material**. Further inquiries can be directed to the corresponding author.
